# Global trends in aspiration pneumonia research since 1980: A bibliometric analysis

**DOI:** 10.1097/MD.0000000000042808

**Published:** 2025-06-06

**Authors:** Akihito Ueda, Kanji Nohara

**Affiliations:** a Department of Internal Medicine, Medical Corporation Toujinkai, Fujitate Hospital, Osaka, Japan; b Graduate School of Pharmaceutical Sciences, Teikyo Heisei University, Tokyo, Japan; c Department of Rehabilitation for Orofacial Disorders, Osaka University Graduate School of Dentistry, Osaka, Japan.

**Keywords:** aspiration pneumonia, bibliometric analysis, elderly pneumonia, pneumonia, research trends

## Abstract

**Background:**

Aspiration pneumonia presents a significant healthcare challenge, particularly in aging societies. Despite extensive research, a comprehensive bibliometric analysis of this field has not been previously conducted. This study aimed to systematically analyze the publication patterns, research collaborations, and thematic trends in aspiration pneumonia research between 1980 and 2024.

**Methods:**

We conducted a bibliometric analysis using data from the Web of Science Core Collection database. The analysis included publication trends, citation patterns, author productivity, and research categories. Network analyses were performed using VOSviewer to visualize international collaborations, author networks, and keyword co-occurrence patterns.

**Results:**

Our analysis of 4668 publications revealed a substantial growth in research output, from 10 publications in 1980 to 371 in 2023. The United States and Japan emerged as the 2 major centers of research output. Analysis of the research categories showed a shift from surgical perspectives during the earlier periods toward more comprehensive medical management approaches in recent years, with General Internal Medicine becoming the leading category between 2010 and 2024. The author collaboration network revealed geographically distinct research clusters with limited cross-regional interactions. Keyword co-occurrence analysis identified 4 major research domains: clinical and epidemiological aspects (41 items), treatment-related approaches (29 items), neurological and swallowing disorders (29 items), and malnutrition.

**Conclusion:**

Our findings demonstrate the evolution of aspiration pneumonia research toward an increasingly multifaceted field that integrates clinical care, rehabilitation, and preventive strategies. The distinct regional patterns in research output suggest opportunities for enhanced international collaboration to advance the current understanding of this condition.

## 1. Introduction

Aspiration pneumonia, resulting from the aspiration of oropharyngeal or gastric contents into the lower respiratory tract, represents a major healthcare challenge.^[1]^ Older adults are at a particularly high risk, owing to reduced swallowing function and multiple comorbidities, making it an increasingly important public health concern in aging societies.^[2,3]^ In Western countries, aspiration pneumonia accounts for 5% to 15% of all pneumonia cases,^[1,3]^ whereas in Japan, it accounts for >60% of hospitalized community-acquired pneumonia cases.^[3,4]^ This trend may reflect certain unique demographic and clinical characteristics of the Japanese population, such as rapid aging and a high prevalence of swallowing disorders.^[5]^

The impact of aspiration pneumonia extends throughout various healthcare systems and societies. It is associated with longer hospital stays, increased healthcare costs, and worse clinical outcomes than non-aspiration pneumonia.^[4]^ Its comprehensive management requires coordinated care across multiple medical disciplines and healthcare settings, ranging from prevention and early detection to acute treatment and rehabilitation. Its multifaceted nature has stimulated significant research interest across a range of medical specialties and scientific fields.

The growing significance of this condition in clinical practice has led to a substantial expansion of research activities focused on it. This diverse body of research continues to shape our understanding of aspiration pneumonia and influence clinical practice. However, despite the expanding literature, a comprehensive bibliometric analysis of this field has not been conducted to date. Understanding the trends and patterns in aspiration pneumonia research through such an analysis may help to guide future research directions and priorities.

Bibliometric analysis, a quantitative method for analyzing academic literature, provides powerful tools for understanding the evolution and current state of scientific fields.^[6]^ For example, in poststroke pneumonia research, 1 bibliometric analysis identified 4 distinct research clusters – risk factors, clinical relevance, mechanisms, and care studies – illustrating how the field has shifted from mechanistic understanding to prevention-focused approaches. It also highlighted strong international collaborations, notably between researchers in China and the United States, that have driven high-impact advancements in the field.^[7]^ Applying this method to aspiration pneumonia research offers an opportunity to potentially uncover similar insights.

This study aimed to systematically analyze the publication patterns, citation networks, and research collaborations related to aspiration pneumonia since 1980. Using bibliometric methods, this analysis sought to provide a deeper understanding of the evolution of the field, identify emerging trends, and suggest directions for future investigations and clinical practice. This study fulfilled these aims through a comprehensive analysis of research development and collaboration patterns, providing insights to guide future research priorities and ultimately contribute to improving the management of this significant healthcare challenge.

## 2. Methods

### 2.1. Data sources and search strategy

We conducted a bibliometric analysis using data from the Web of Science Core Collection database through a search conducted on January 2, 2025, that covered literature spanning the years 1980 to 2024. Papers published in 2025 were excluded. A topic search was performed using the term “aspiration pneumonia.” We included articles, review articles, and research letters as document types, assuming that the latter, an often overlooked publication type, may also contain significant contributions to the field. To maintain a focus on human-related studies, research categories such as veterinary sciences and zoology were excluded.

### 2.2. Data extraction and processing

The bibliographic records were exported in full-record format, including author names, title, abstract, keywords, journal, publication year, citations, and institutional affiliations. Duplicate records were removed to ensure data accuracy. To standardize the data, author names were checked for variations, institution names were harmonized, and country/region names were unified according to standard geographical classifications. The processed data were then prepared for analysis and visualization.

### 2.3. Bibliometric analysis

This bibliometric analysis followed established guidelines,^[6]^ complemented by co-citation analysis principles introduced by Small.^[8]^ The key indicators analyzed included:

1. Publication trends: annual publication output over time;
2. Citation analysis: the most highly-cited publications and their characteristics;
3. Author analysis: number of publications per author;
4. Institution and country analysis: geographic distribution of research output and collaboration networks;
5. Keyword analysis: temporal changes in research topics and themes;
6. Research field analysis: distribution of publications across the various Web of Science categories.

Our citation analysis identified the most influential articles in the field over the study period of 1980 to 2024, and our author analysis revealed the most productive researchers and their publication patterns. We examined the distribution of research categories over the same period through our research field analysis. To characterize the temporal changes in research categories, we divided the study period into 3 distinct periods: 1980 to 1994, 1995 to 2009, and 2010 to 2024.

### 2.4. Network analysis and visualization

Network analysis and visualization were conducted using VOSviewer version 1.6.20 (Centre for Science and Technology Studies, Leiden University, Leiden, Netherlands), a widely used software tool for bibliometric mapping and analysis.^[9]^ This approach enabled the identification of research clusters, collaboration patterns, and major thematic areas within the dataset. We created and analyzed 3 distinct networks:

1. International collaboration network: to visualize country-wise distributions of research output and collaboration patterns;
2. Author collaboration network: to map co-authorship patterns among researchers;
3. Keyword co-occurrence network: to analyze the relationships between research topics based on keyword co-occurrence.

### 2.5. Ethics statement

This study did not require ethical approval as it analyzed only publicly available data from the Web of Science Core Collection database. No human subjects, animals, or personally identifiable information were involved in this research.

## 3. Results

### 3.1. Search results and publication trends

**Figure 1.**
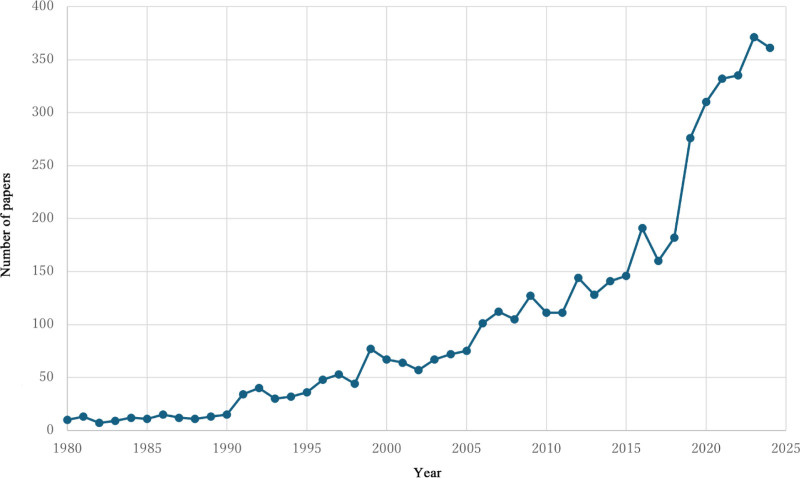
Annual publication trends in aspiration pneumonia research.

Our search of the Web of Science Core Collection database yielded 4670 records. Two duplicate entries were removed, leaving 4668 unique publications. Figure 1 shows the annual publication trends in aspiration pneumonia research. The annual publication count increased from 10 in 1980 to 371 in 2023.

### 3.2. Author productivity and citation impact

Our analysis of publication output by author (Table 1) revealed that Japanese publications represented the majority in terms of volume. Our analysis of citation data (Table 2) revealed that review articles accounted for the majority of the most cited articles, particularly in high-impact medical journals. Among these highly-cited review articles, Yoneyama et al original research paper,^[10]^ which demonstrated the effectiveness of oral care in preventing pneumonia, was the only Japanese-authored report in the top 10 citations.

**Table 1:** Most productive authors in aspiration pneumonia research (1980–2024).

Rank	Author	Country	Number of publications
1	Ebihara, Satoru	Japan	27
2	Clave, Pere	Spain	26
3	Ebihara, Takae	Japan	22
4	Troche, Michelle S	USA	21
5	Komiya, Kosaku	Japan	19
6	Mukae, Hiroshi	Japan	18
7	Teramoto, Shinji	Japan	17
7	Brook, Itzhak	USA	17
9	Fushimi, Kiyohide	Japan	16
9	Yoshimatsu, Yuki	Japan	16
9	Momosaki, Ryo	Japan	16

**Table 2:** Most cited articles in aspiration pneumonia research (1980–2024).

Rank	Authors	Title	Journal	Year	Citations
1	Marik	Primary care: aspiration pneumonitis and aspiration pneumonia.	New England Journal of Medicine	2001	964
2	Wong et al	Clinical and pathophysiological overview of acinetobacter infections: a century of challenges	Clinical Microbiology Reviews	2017	724
3	Bernard et al	High-dose corticosteroids in patients with the adult respiratory distress syndrome	New England Journal of Medicine	1987	651
4	Marik et al	Aspiration pneumonia and dysphagia in the elderly	Chest	2003	596
5	Finucane et al	Tube feeding in patients with advanced dementia: a review of the evidence	JAMA – Journal of the American Medical Association	1999	586
6	Langmore et al	Predictors of aspiration pneumonia: how important is dysphagia?	Dysphagia	1998	560
7	Sura et al	Dysphagia in the elderly: management and nutritional considerations	Clinical Interventions in Aging	2012	523
8	Westendorp et al	Poststroke infection: a systematic review and meta-analysis	BMC Neurology	2011	514
9	Yoneyama et al	Oral care reduces pneumonia in older patients in nursing homes	Journal of the American Geriatrics Society	2002	469
10	Zangrillo et al	A meta-analysis of complications and mortality of extracorporeal membrane oxygenation	Critical Care and Resuscitation	2013	445

### 3.3. Research categories

Our overall analysis of research output (Table 3) by Web of Science categories across the entire study period revealed that General Internal Medicine had the most publications (660), followed by Surgery (501), Clinical Neurology (469), Otorhinolaryngology (410), and Respiratory System (328). Among all of the categories analyzed, the presence of Geriatrics Gerontology (6th, 323), Rehabilitation (13th, 158), and Dentistry Oral Surgery Medicine (14th, 154) in the top rankings demonstrated the multidisciplinary nature of aspiration pneumonia research. Table 4 shows the top 15 categories over the 3 time periods of 1980 to 1994, 1995 to 2009, and 2010 to 2024. While Surgery ranked first in the 2 earlier periods, General Internal Medicine led the most recent period (2010–2024). Notable changes included Geriatrics Gerontology moving from being absent in 1980 to 1994, to eighth place in 1995 to 2009 and fifth place in 2010 to 2024, followed by the emergence of Rehabilitation and Dentistry Oral Surgery Medicine among the top categories during the recent period.

**Table 3:** Web of science categories in aspiration pneumonia research (1980–2024).

Rank	Category	Number of publications
1	General Internal Medicine	660
2	Surgery	501
3	Clinical Neurology	469
4	Otorhinolaryngology	410
5	Respiratory System	328
6	Geriatrics Gerontology	323
7	Gastroenterology Hepatology	279
8	Pediatrics	228
9	Neurosciences	200
10	Critical Care Medicine	195
11	Pharmacology Pharmacy	184
12	Infectious Diseases	164
13	Rehabilitation	158
14	Dentistry Oral Surgery Medicine	154
15	Gerontology	153

**Table 4:** Top web of science categories in aspiration pneumonia research (1980–1994, 1995–2009, and 2010–2024).

Rank	Category (1980–1994)	Category (1995–2009)	Category (2010–2024)
1	Surgery	Surgery	General Internal Medicine
2	General Internal Medicine	Clinical Neurology	Clinical Neurology
3	Pediatrics	General Internal Medicine	Otorhinolaryngology
4	Respiratory System	Otorhinolaryngology	Surgery
5	Anesthesiology	Respiratory System	Geriatrics Gerontology
6	Gastroenterology Hepatology	Pediatrics	Respiratory System
7	Critical Care Medicine	Critical Care Medicine	Gastroenterology Hepatology
8	Radiology Nuclear Medicine Medical Imaging	Geriatrics Gerontology	Neurosciences
9	Clinical Neurology	Gastroenterology Hepatology	Pharmacology Pharmacy
10	Infectious Diseases	Pharmacology Pharmacy	Dentistry Oral Surgery Medicine
11	Pharmacology Pharmacy	Radiology Nuclear Medicine Medical Imaging	Rehabilitation
12	Microbiology	Neurosciences	Infectious Diseases
13	Cardiac Cardiovascular Systems	Gerontology	Pediatrics
14	Immunology	Rehabilitation	Gerontology
15	Otorhinolaryngology	Infectious Diseases	Medicine Research Experimental

### 3.4. International collaboration network

**Figure 2.**
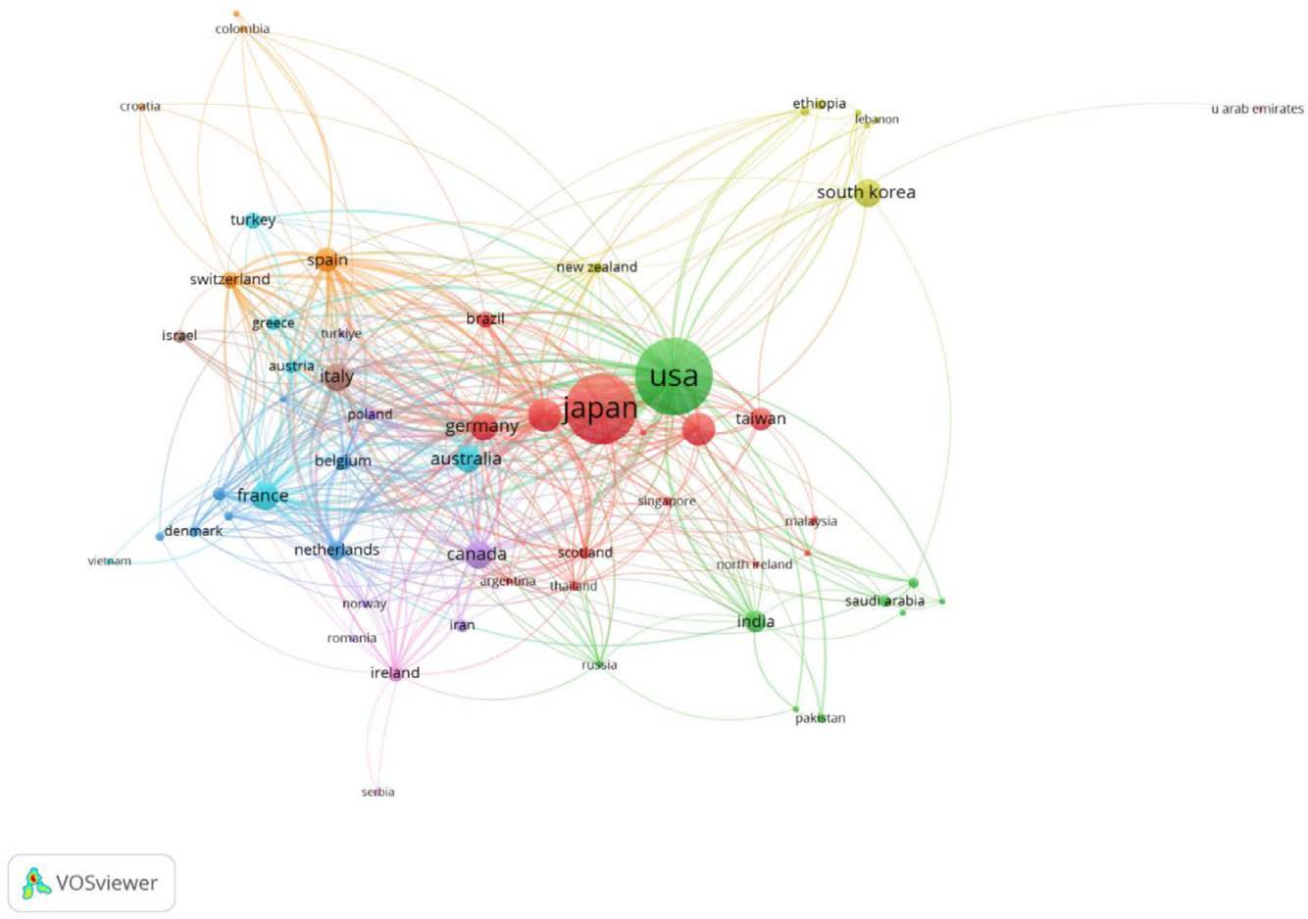
International collaboration network in aspiration pneumonia research. Node sizes represent the number of publications. Edge width indicates the strength of collaboration between countries.

The international collaboration network (Fig. 2) identified 2 major centers of research output: the USA (1277 publications, 47,817 citations) and Japan (1078 publications, 15,729 citations). Other significant contributors included the People’s Republic of China (242 publications, 2714 citations), England (233 publications, 8492 citations), and Germany (178 publications, 5706 citations).

### 3.5. Author collaboration network

**Figure 3.**
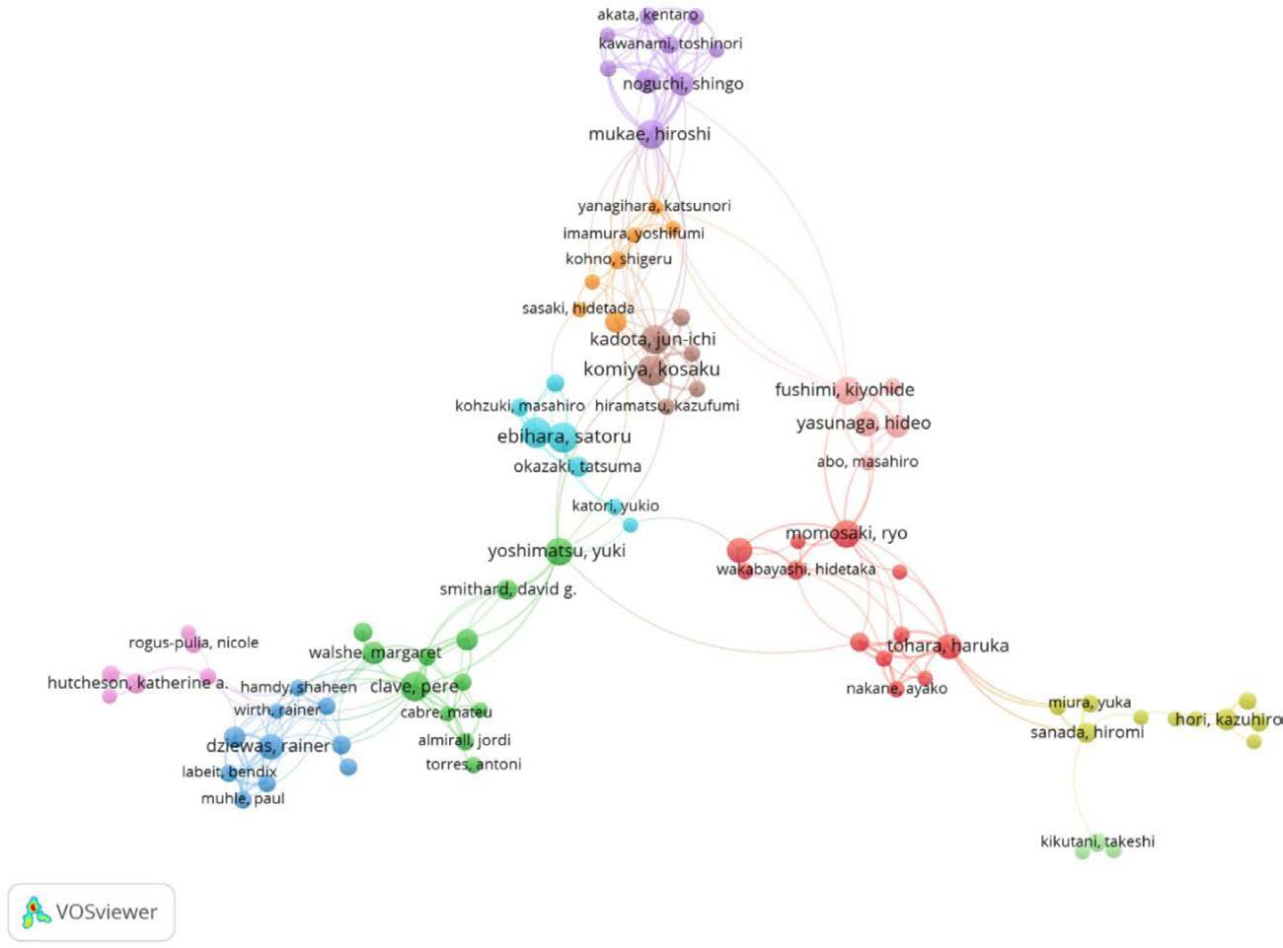
Author collaboration network in aspiration pneumonia research. Node size represents the number of publications per author. Edge width indicates the frequency of co-authorship.

The author collaboration network (Fig. 3) revealed distinct research clusters within Japan, as well as a separate network formed by Western researchers. There was minimal interconnection or collaboration between these networks, highlighting a lack of interaction between Japanese and Western research communities.

### 3.6. Keyword co-occurrence network

**Figure 4.**
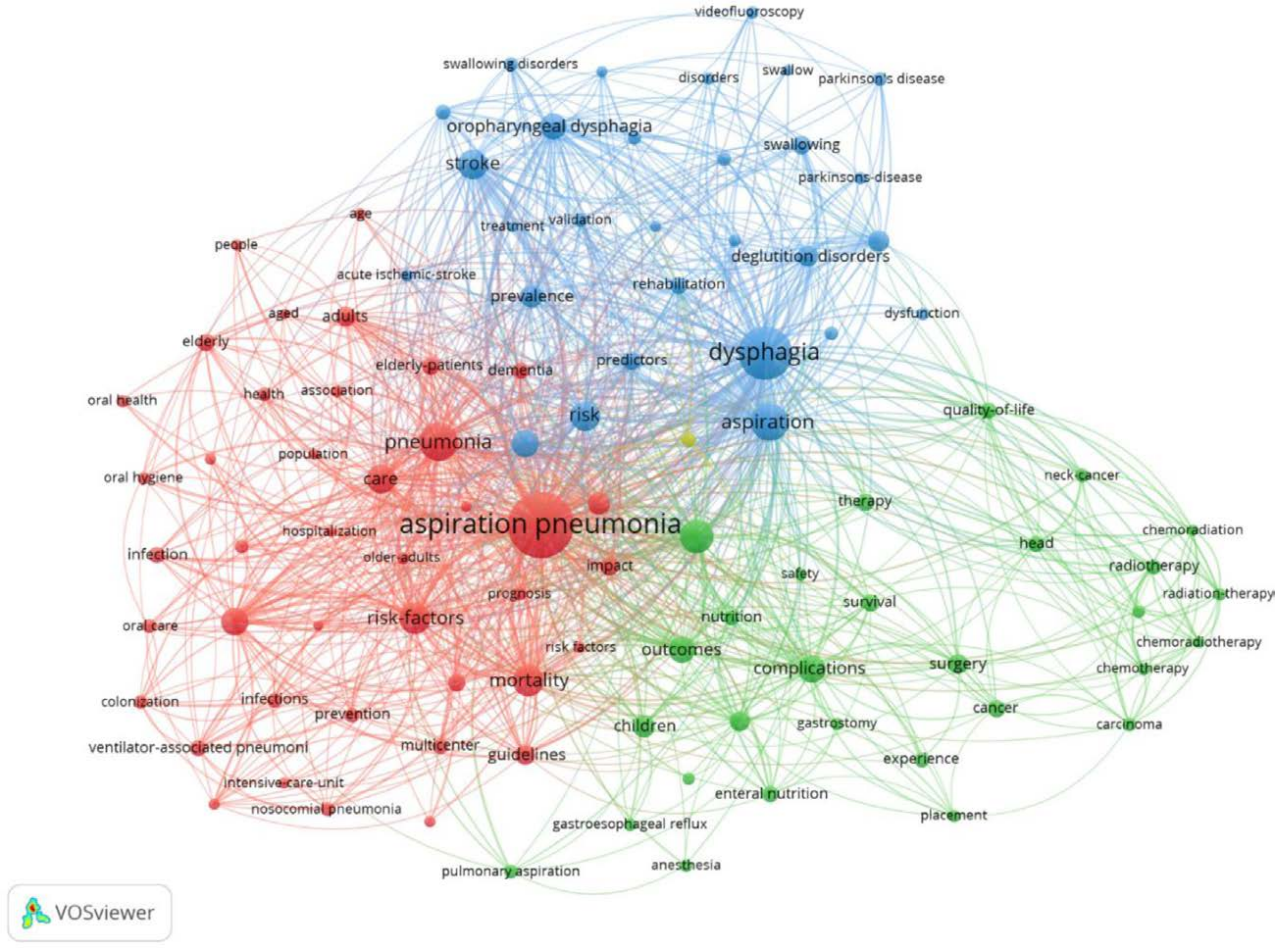
Keyword co-occurrence network in aspiration pneumonia research. Node size represents the frequency of keyword usage. Edge width indicates the frequency of co-occurrence.

A network analysis of keyword co-occurrence was performed using the top 100 keywords with the strongest co-occurrence links among all of the keywords indexed in the Web of Science database. The visualization (Fig. 4) identified 4 distinct clusters: clinical and epidemiological aspects (41 items), treatment-related terms (29 items), neurological and swallowing disorders (29 items), and malnutrition as a standalone cluster. The temporal distribution is represented by color gradients.

## 4. Discussion

### 4.1. Publication trends

Aspiration pneumonia research has grown substantially over the past 45 years. This likely reflects a growing recognition of this condition as a significant healthcare challenge, particularly in aging societies.^[3]^ As populations age, pneumonia has become a leading cause of death in older adults, with aspiration often being a major contributing mechanism.^[4,11]^ This recognition may have driven increased research into risk factors, disease prevention, and management strategies.^[3,10,12–14]^

### 4.2. Geographic distribution and research impact

Our international collaboration network analysis (Fig. 2) identified 2 major centers of research output: the USA (1277 publications, 47,817 citations) and Japan (1078 publications, 15,729 citations). These 2 countries substantially outperformed all other nations in terms of publication volume. This publication pattern differs notably from that of other respiratory conditions; for instance, in poststroke pneumonia research, Chinese institutions have shown comparable output to those in the U.S.^[7]^ The high research output from Japan may reflect its rapidly aging population and the consequent high prevalence of aspiration pneumonia.

Our analysis of citation patterns (Table 2) reveals that review or review-like articles constituted a substantial portion of the most cited articles. The most cited article was Marik review in the New England Journal of Medicine (964 citations),^[1]^ which made significant contributions by distinguishing aspiration pneumonitis from aspiration pneumonia and providing a comprehensive pathophysiological framework for it. Among the original research reports, Yoneyama et al randomized controlled trial (469 citations, ranked ninth) stands out as a significant contribution that demonstrated the effectiveness of oral care for preventing pneumonia.^[10]^ That study significantly influenced clinical approaches to pneumonia prevention.

The pattern of research productivity revealed notable regional characteristics. The concentration of highly productive researchers in Japan contrasts with the more distributed pattern seen in Western countries, particularly the USA. Despite leading in total publications, researchers in the USA tended to show more moderate individual publication numbers. This discrepancy between total publications and individual productivity rankings may reflect differences in authorship practices and research team structures between regions. Japanese research groups may tend to maintain focused research lines concerning aspiration pneumonia, resulting in higher individual publication counts, while research contributions in the USA might come from a broader range of researchers and teams working across various aspects of the condition.

### 4.3. Research communities and collaboration

The author collaboration network (Fig. 3) revealed a significant division in the aspiration pneumonia research community. Japanese researchers formed distinct research clusters with dense internal connections, while Western ones constituted another separate collaborative network. This division manifested in both publication patterns and research priorities.

Despite the increasing research output from both regions over time, collaboration patterns remain largely unchanged, with minimal interaction between Japanese and Western research communities. These distinct research patterns may be related to the differing incidence rates of aspiration pneumonia reported across regions. In Western countries, it is estimated that 5% to 15% of pneumonia cases involve aspiration pneumonia,^[1,3]^ whereas it has been reported to account for > 60% of hospitalized community-acquired pneumonia cases in Japan.^[4,15]^

Several factors may contribute to this observed lack of collaboration between Japanese and Western researchers. First, language barriers likely play a significant role. While Japanese researchers increasingly publish in English, everyday communication and the conceptual stages of collaborative research design may be hindered by language differences. This is particularly evident when discussing complex clinical concepts and cultural contexts related to elderly care.

Second, differences in research and publication cultures may also be a contributing factor. Japanese medical research tends to emphasize long-term care and prevention, reflecting the context of the Japanese healthcare system and aging society. In contrast, Western (particularly U.S.) research often focuses more on short-term treatment outcomes and clinical trials. For example, Japanese studies frequently emphasize preventive approaches such as oral care and swallowing rehabilitation, while Western research shows a stronger trend toward studies on antibiotic therapy and acute management. These divergent research priorities may limit opportunities for collaborative projects.

Funding mechanism disparities also present a significant barrier. Japanese research funding is primarily provided by domestic institutions (Ministry of Education, Culture, Sports, Science and Technology; Ministry of Health, Labour and Welfare, etc) with limited explicit support for international collaborative projects. Similarly, Western funding agencies tend to prioritize domestic research. Additionally, time differences and administrative variations in collaborative research design and application may impede international cooperation.

To overcome these barriers, several specific strategies could be considered:

1. Organization of regular international symposia: hosting regular international conferences specifically focused on aspiration pneumonia would increase opportunities for direct interaction and network formation among researchers. Alternating between Japanese and Western locations would encourage participation from both regions.
2. Enhancement of researcher exchange programs: expanding short and long-term exchange programs for early-career researchers would promote experience in different research environments. Exchange of clinically oriented researchers would be particularly valuable for understanding differences in diagnostic and treatment approaches.
3. International standardization of diagnostic criteria and research methodologies: efforts to internationally standardize diagnostic criteria and research methodologies for aspiration pneumonia would enhance the comparability of research results and strengthen the foundation for collaborative research. Integration of different diagnostic approaches between Japan and the West is a particularly important challenge.

Implementation of these strategies requires cooperation from research institutions, professional medical societies, and funding agencies. Strengthening international research collaboration would play an essential role in deepening our understanding of the true prevalence, risk factors, and effective prevention and treatment strategies for aspiration pneumonia.

One reason for this discrepancy may be the differences in diagnostic approaches between Japan and Western countries. As we previously reported,^[16]^ pneumonia that occur in patients with risk factors for aspiration in Japan are often diagnosed as aspiration pneumonia without specific supporting evidence. This widespread diagnostic practice stems from Teramoto et al criteria (cited 318 times, ranked 23rd),^[4]^ which became widely adopted in Japan after their 2008 report of a high incidence of aspiration pneumonia in the country. Since then, Japanese clinicians have frequently diagnosed pneumonia in patients with aspiration risk factors as aspiration pneumonia without considering the characteristic imaging findings of the condition. In our recent study,^[17]^ we demonstrated that this diagnostic approach may lead to overdiagnosis of aspiration pneumonia. Addressing these diagnostic uncertainties through enhanced international collaboration and establishing unified diagnostic criteria would facilitate a more accurate assessment of the true incidence of aspiration pneumonia.

In this context, Yoshimatsu, Yuki represents a researcher who might serve as a bridge between these communities, maintaining collaborative ties with both Japanese and Western researchers. This bridging role is demonstrated in his team’s recent publications, which span Japanese and European institutions – including a collaboration with researchers from European institutions to compile a systematic review concerning the diagnostic criteria for aspiration pneumonia,^[18]^ and unite multiple Japanese institutions while maintaining UK affiliations for a meta-analysis on treatment approaches.^[19]^ Such bridging roles may be crucial for future efforts to harmonize diagnostic criteria and research approaches across regions.

### 4.4. Research categories and field evolution

The distribution of research across the Web of Science categories revealed significant shifts in research focus over the 45-year period, reflecting an evolving understanding of the condition’s nature. Surgery’s decline from the top rank during the earlier periods (i.e., 1980–1994 and 1995–2009) to fourth place in 2010 to 2024 may reflect the field’s evolution from a focus on surgical complications and feeding interventions such as gastrostomy to an increased understanding of aspiration pneumonia as a complex disorder that may require coordinated care across multiple specialties. The emergence of General Internal Medicine as the leading category from 2010 to 2024 highlights this shift. Notably, while aspiration pneumonia is fundamentally a respiratory infection, neither Respiratory System nor Infectious Diseases consistently ranked as the top categories, with General Internal Medicine instead taking the leading position. This suggests that the condition’s management extends beyond traditional pneumonia treatment. Similarly, Anesthesiology’s presence in the fifth rank between 1980 to 1994, then subsequent disappearance from the top 15 during the later periods, may indicate a conceptual shift in the field – from acute aspiration of gastric contents that was categorized as “aspiration pneumonitis” in Marik review paper (exemplified by Mendelson syndrome,^[20]^ characterized by chemical pneumonitis and acute lung injury),^[1]^ toward the current understanding of aspiration pneumonia as primarily resulting from the chronic microaspiration of oral bacteria.

The rise of the Clinical Neurology, Neurosciences, and Otorhinolaryngology fields among the more recent rankings highlights an increased attention to underlying mechanisms and specialized care approaches, particularly in terms of addressing swallowing disorders. The decline in the ranking of the Pediatrics field, from third place in 1980 to 1994 to 13th in 2010 to 2024, reflects the field’s reorientation toward geriatric populations. This aligns with the growing recognition of aspiration pneumonia as a predominantly age-related condition. This shift is further evidenced by Geriatrics Gerontology’s rise in popularity, from being absent in 1980 to 1994 to eighth place in 1995 to 2009 and finally fifth place in 2010 to 2024. The emergence of the Rehabilitation and Dentistry Oral Surgery Medicine fields in recent years emphasized the field’s evolution toward preventive and comprehensive care strategies, acknowledging the importance of swallowing rehabilitation and oral care in the management of aspiration pneumonia.

### 4.5. Evolution of research themes

Our keyword co-occurrence network analysis (Fig. 4) identified 4 distinct clusters that characterize the current research landscape. The largest cluster encompasses clinical and epidemiological aspects (41 items), including core terms such as “pneumonia,” “mortality,” and “infection.” One notable finding from the network visualization was that “dysphagia” appeared as a node that was comparable in size to “aspiration pneumonia,” suggesting its fundamental role in the disease process. The prominence of dysphagia-related research, represented in the neurological and swallowing disorders cluster (29 items), reflects its significant role in the pathophysiology of aspiration pneumonia. The treatment-related cluster (29 items) focused on interventional approaches such as “surgery,” “chemotherapy,” and “complications,” with particular emphasis on treatment-related complications and nutritional management. The connections between these terms suggest a need for attention to various medical interventions and their associated risks. The emergence of malnutrition as a distinct cluster demonstrated its significant role in disease outcomes, showing strong connections to both the treatment-related and swallowing disorder clusters. These interconnected clusters highlight how aspiration pneumonia research has evolved into a multifaceted field, emphasizing comprehensive care that addresses multiple risk factors. This structure has significant implications for research funding strategies and integrated educational programs for healthcare professionals. However, further studies are warranted to determine the optimal balance between these domains.

The evolution of research themes and analysis of keyword co-occurrence patterns revealed in this study provide important implications for clinical practice. In particular, the interconnections between the 4 major clusters highlight the need for comprehensive approaches to the prevention and management of aspiration pneumonia.

#### 4.5.1. Implications from the clinical/epidemiological aspects cluster

The epidemiological patterns and clinical features emphasized in this cluster directly relate to the development of risk-stratification tools. For instance, the close association of keywords such as “elderly,” “dementia,” and “stroke” shown in the VOSviewer analysis suggests that screening tools combining these factors may be effective. Clinicians should adopt more aggressive preventive strategies for patients with multiple risk factors (e.g., adult patients with a history of dementia and stroke), including regular swallowing function assessments and early intervention programs.

#### 4.5.2. Implications from the neurological and swallowing disorders cluster

The findings from this cluster emphasize that the assessment and management of dysphagia are central to aspiration pneumonia prevention. In clinical practice, the strong connections between keywords such as “videofluoroscopy,” “swallowing function,” and “dysphagia” in the VOSviewer network highlight the importance of regular swallowing assessments. Swallowing evaluations should be incorporated into standard care protocols, particularly for patients with stroke or neurodegenerative diseases. Early swallowing training programs in collaboration with rehabilitation specialists are also recommended.

#### 4.5.3. Implications from the treatment-related approaches cluster

Analysis of this cluster highlights the complexity of therapeutic interventions and the need for attention to potential complications. In clinical practice, decisions regarding nutritional interventions, such as gastrostomy, require a careful assessment of aspiration risk and nutritional status. The close association of “complications” and “prognosis” in the keyword network emphasizes the importance of a balanced decision-making process that considers both short-term and long-term risks of interventions. The appropriate use of antibiotics and the integration of approaches combining swallowing rehabilitation and nutritional management are also important.

#### 4.5.4. Implications from the malnutrition cluster

The existence of malnutrition as a distinct cluster indicates that assessment and management of nutritional status play a central role in preventing and treating aspiration pneumonia. In clinical practice, regular nutritional assessment and early intervention are recommended for all high-risk patients. For patients at risk of both dysphagia and malnutrition, individualized meal plans and texture modifications developed through collaboration between dietitians and speech-language pathologists are effective.

#### 4.5.5. Implications for interdisciplinary care models

The interconnections among these 4 clusters demonstrate that multidisciplinary collaborative approaches are essential for effective management of aspiration pneumonia. Specifically, the formation of comprehensive care teams including internists, geriatricians, neurologists, dentists, speech-language pathologists, dietitians, and nurses is recommended. Such team approaches can integrate knowledge and skills from each specialty area to develop individualized prevention and management plans, particularly for adult patients with complex health issues.

#### 4.5.6. Implications for prevention strategies

The importance of preventive interventions such as “oral care,” “swallowing rehabilitation,” and “positioning” revealed in keyword analysis indicates the need to systematically incorporate these methods into clinical practice. Particularly in elderly care facilities and home care settings, including these prevention strategies in routine care protocols could significantly reduce the incidence of aspiration pneumonia.

The effective implementation of these clinical implications requires ongoing education for healthcare providers and the development of evidence-based guidelines. Strategies to facilitate knowledge transfer from research to clinical practice include developing clinical practice guidelines, implementing quality improvement initiatives, and utilizing clinical decision support tools.

### 4.6. Future research directions

The current state of the field suggests several promising directions for future research. First, exploring and harmonizing diagnostic approaches across regions may help to foster a better understanding of the true epidemiology of aspiration pneumonia. Second, considering the limited representation of Japanese research among highly-cited papers despite the significant research activity in Japan, a greater emphasis on international collaborative research may help to bridge this gap. Recent trends in preventive approaches, particularly in the areas of oral care and swallowing rehabilitation, indicate promising areas for future investigations into more effective preventive strategies for high-risk older populations.

## 5. Limitations

Some limitations should be considered when interpreting our findings. First, our analysis relied solely on the Web of Science Core Collection, which may not capture all relevant publications. This is particularly true for Japanese research, as significant findings in Japanese-language journals are often excluded, potentially underestimating Japan contributions. Additionally, our topic-based search strategy for “aspiration pneumonia” aimed to capture the field’s overall landscape, but may have included papers of limited relevance. Furthermore, regarding publications from 2024, some articles may not have been indexed at the time of our data collection (January 2, 2025), due to indexing delays.

## 6. Conclusions

Our bibliometric analysis revealed a substantial growth in aspiration pneumonia research over the past 45 years. The research output has been dominated by 2 regions, the United States and Japan, with limited cross-regional collaboration. Our analysis demonstrated the evolution in research focus across medical specialties and growing emphasis on preventive approaches. Enhanced international research collaboration may help to advance our current understanding of aspiration pneumonia and improve patient care for the condition globally.

## Acknowledgments

We would like to thank Editage (www.editage.com) for their assistance with English-language editing.

## Author contributions

**Conceptualization:** Akihito Ueda.

**Data curation:** Akihito Ueda.

**Formal analysis:** Akihito Ueda, Kanji Nohara.

**Funding acquisition:** Kanji Nohara.

**Investigation:** Akihito Ueda.

**Methodology:** Akihito Ueda.

**Project administration:** Kanji Nohara.

**Resources:** Akihito Ueda.

**Software:** Akihito Ueda.

**Validation:** Kanji Nohara.

**Visualization:** Akihito Ueda.

**Writing – original draft:** Akihito Ueda.

**Writing – review & editing:** Kanji Nohara.
